# Clinical and Paraclinical Peculiarities of SARS-CoV-2 and Influenza Infections in Children: A Comparative Study

**DOI:** 10.3390/life15050784

**Published:** 2025-05-14

**Authors:** Maria Oana Săsăran, Carmen Viorica Muntean, Andreea Bianca Stoica, Carmen Schwesig, Anca Meda Văsieșiu, Anca Doina Pleșca, Cristina Oana Mărginean

**Affiliations:** 1 Department of Pediatrics 3, “George Emil Palade” University of Medicine, Pharmacy, Science, and Technology of Targu Mures, Gheorghe Marinescu Street No. 38, 540136 Targu Mures, Romania; oanam93@yahoo.com; 2 Department of Pediatrics 1, “George Emil Palade” University of Medicine, Pharmacy, Science, and Technology of Targu Mures, Gheorghe Marinescu Street No. 38, 540136 Targu Mures, Romania; andreeabstoica9@gmail.com (A.B.S.); marginean.oana@gmail.com (C.O.M.); 3 Department of Internal Medicine, Weser-Egge Clinic-St. Josef Hospital Bad Driburg, Elmarstrasse No. 38, 33014 Bad Driburg, Germany; c.schwesig@gmx.net; 4 Faculty of Medicine in English, “George Emil Palade” University of Medicine, Pharmacy, Science, and Technology of Targu Mures, Gheorghe Marinescu Street No. 38, 540136 Targu Mures, Romania; 5 Department of Infectious Disease, “George Emil Palade” University of Medicine, Pharmacy, Science, and Technology of Targu Mures, Gheorghe Marinescu Street No. 38, 540136 Targu Mures, Romania; anca-meda.vasiesiu@umfst.ro; 6 Department of Pediatrics, Carol Davila University of Medicine and Pharmacy, Boulevard Eroii Sanitari No. 8, 050474 Bucharest, Romania; doinaplesca@yahoo.com

**Keywords:** SARS-CoV-2 infection, influenza infection, hematological parameters, children

## Abstract

Background: SARS-CoV-2 and influenza can present with similar clinical pictures in children, with symptoms and paraclinical particularities which might aid in the differentiation of the two entities and which can be suggestive of various complications. The present study aims to identify clinical and paraclinical differences between pediatric SARS-CoV-2 and influenza infection and to assess the utility of hematological parameters for prediction of their related complications. Methods: In this study, 266 children were retrospectively enrolled, divided into two groups: 129 children diagnosed with SARS-CoV-2 infection and 137 children infected with influenza. In each case, particular symptoms were recorded, as well as hospitalization duration, pediatric intensive care unit (PICU) admission or O_2_ supplementation requirement. Parameters of the hemoleucogram and biochemistry parameters were also collected for comparative assessment. Results: SARS-CoV-2 infections were more commonly associated with digestive symptoms, whereas influenza infections implied longer hospital stays and higher likelihood of PICU admission necessity. Monocytes and lymphocyte/monocyte ratios (LMRs) were significantly higher in the SARS-CoV-2 group (*p* < 0.01, *p* = 0.02). Several hematological parameters, such as neutrophil/lymphocyte ratios, correlated with hospitalization duration in SARS-CoV-2 and influenza B infections (*p* < 0.01, *p* = 0.01), whereas LMR was predictive of respiratory distress (*p* = 0.02) in the same study groups. Conclusions: According to the study, monocyte levels and LMR can aid in the distinction of pediatric SARS-CoV-2 and influenza infections and LMR and NLR can be used particularly as predictors of complicated course of these infections.

## 1. Introduction

Influenza and SARS-CoV-2 infections cause similar clinical pictures and are hard to distinguish in clinical practice in the absence of etiological testing. Fever and cough are the most frequent symptoms which commonly occur in both types of viral infections in children, especially in influenza infections, whereas rhinorrhea seems to be more customary in influenza infections [1]. In-hospital mortality is believed to be similar between the two infections, with a similar percentage of children requiring mechanical ventilation, according to some authors [2]. Some other studies report higher pediatric intensive care unit (PICU) hospitalizations for SARS-CoV-2 infections, due to mechanical ventilation necessity, but acknowledge that mortality rates are neglectable for both infections [1,3]. Still, particular forms of influenza, such as H1N1 influenza, are accountable for a significantly higher duration of hospitalization than SARS-CoV-2 [4]. Pneumonia seems to be a complication that occurs more commonly in children with influenza [3]. In outpatient settings, SARS-CoV-2 infection is accountable for more prolonged symptom evolution, as highlighted by a study comparatively analyzing household transmission of the aforementioned virus and influenza [5].

The COVID-19 pandemic and the lockdown measures that were inflicted led to a temporary decline in the transmission of respiratory infections, with resurgence of commonly encountered infections after the cessation of restrictive measures. Hence, a pediatric study conducted in China has found the lowest positivity rates for influenza A, influenza B, adenovirus and respiratory syncytial virus in 2020, followed by a significant increase in etiological diagnoses of the same infection in 2022 [6]. Nevertheless, actual studies focus on the behavior of influenza infections before and after the pandemic. Higher incidence of the infection has been reported in individuals over 3 years of age through a more accentuated spread of the viral strains, which translates into more cases of pneumonia, as the influenza viruses maintain the same percentages of complications as before the pandemic [7]. Hence, the analysis of the severity of viral respiratory tract infections in children requires further comparative studies, which include the post-pandemic period.

Accurate diagnosis of infectious etiology frequently requires nucleic viral acid testing, which often involves costly expenses. Hence, current research tries to figure out possibilities to make a distinction between various respiratory tract infections, based on clinical and paraclinical data [8]. Hence, hematological parameters, as well as inflammatory indexes derived from their values, have been recently used for differential diagnosis of viral and bacterial infections, as well as possible predictors of adverse outcomes and complications [9,10]. Newly applied hematological ratios, such as NLR and PLR, could allegedly help differentiate influenza from viral infections [10]. In a comparative study, SARS-CoV-2 infected patients were distinguished from those with influenza B infections through higher leukocyte counts, neutrophilia, and lower lymphocyte counts. Influenza B infections seemed to be accompanied by higher absolute red blood cell numbers when compared to SARS-CoV-2 subjects [8]. One pediatric study also highlighted that eosinophil and monocyte counts can potentially distinguish between SARS-CoV-2 and influenza infections [11]. Laboratory parameters, including hematological markers, remain important for the monitoring of disease course and as predictors of disease severity in both SARS-CoV-2 and influenza infections, as suggested by several lines of evidence [12,13]. Hence, current research has focused on the role of easily accessible parameters, including those of complete blood count (CBC), for the early prediction of adverse outcomes in viral respiratory tract infections.

The present study aimed to compare clinical and paraclinical characteristics of SARS-CoV-2 and influenza infections in a pediatric population, while focusing on the importance of hematological parameters for distinction between the two infections and for prediction of associated complications.

## 2. Materials and Methods

### 2.1. Study Population

The current research involved a retrospective, observational study, which enrolled a pediatric population, aged between 0 and 15 years of age, admitted for SARS-CoV-2 and influenza infections between January 2020 and December 2024 in a tertiary pediatric referral center in Târgu Mureș, Romania. The study population consisted of 266 children divided as follows: 129 children infected with SARS-CoV-2 and 137 children infected with Influenza (101 patients with type A Influenza and 36 patients with type B Influenza). The diagnosis was based upon real time polymerase chain reaction testing (RT-PCR) or rapid viral antigen testing, using a nose and/or throat swab, according to the manufacturer’s indication. RT-PCR tests were initially performed at the beginning of the SARS-CoV-2 pandemic, when rapid viral antigen tests were not available, and afterwards in patients with suggestive clinical pictures of SARS-CoV-2, but with a negative rapid viral antigen tests. Rapid respiratory viral antigen testing included combined diagnostic kits for SARS-CoV-2, respiratory syncytial virus (RSV), influenza A and influenza B (CerTest Biotec, Zaragoza, Spain). Patients with known underlying, chronic disorders, such as hematological diseases, lymphoproliferative disorders, oncologic diagnoses, cardiac malformations with hemodynamic impact, neurologic disorders or genetic syndromes, were excluded from the study. Moreover, we excluded those cases with incomplete records of the parameters of interest. Furthermore, respiratory viral co-infections were ruled out, including influenza and SARS-CoV-2 co-infections, as well as SARS-CoV-2 infections with adverse outcome that complied with a multisystem inflammatory syndrome, according to the latest definition standardized by the Centers for Disease Control and Prevention (CDC) [14].

### 2.2. Clinical and Paraclinical Data

The clinical symptom pattern was retrieved from the patients’ charts, while focusing on symptoms such as fever, nasal obstruction, dysphagia, dysphonia, cough, otalgia, vomiting, diarrhea or abdominal pain. In each case, hospitalization duration was recorded, along with saturation levels at the time of admission, as well as eventual admission in the pediatric intensive care unit (PICU) and subsequent length of stay. The following paraclinical data were gathered: absolute numbers for parameters of the complete blood count (CBC), aspartate aminotransferase (AST), alanine aminotransferase (ALT), urea, creatinine, lactate dehydrogenase (LDH) and C reactive protein (CRP) levels. The absolute value for each parameter of CBC was recorded and the following subsequent ratios were calculated through division of these numbers: lymphocyte to monocyte ratio (LMR), platelet to lymphocyte ratio (PLR) and neutrophil to lymphocyte ratio (NLR). Moreover, the presence of complications, such as dehydration, respiratory distress, febrile seizures and chest ray abnormalities, compatible with pneumonia was also recorded.

### 2.3. Statistical Data

The statistical analysis was performed with the help of GraphPad Prism, version 10.4.1. Descriptive statistics were conducted for quantitative variables, which led to the calculation of data such as median and/or mean ± standard deviation (SD). For assessment of data distribution patterns, the Kolmogorov–Smirnov normality test was applied. For mean comparison of two unpaired data sets, the unpaired t test with Welch’s correction or the Mann–Whitney test was applied, depending on the compliance of the analyzed data with a Gaussian distribution pattern. Multiple data sets were compared with the help of Brown–Forsythe and the Welch ANOVA test for data following a Gaussian distribution, whereas the Kruskal–Wallis test was applied for multiple mean comparison of non-Gaussian distributed data. Contingency tables, which encompassed qualitative variables, were analyzed with the Chi square test and, for each analysis, a *p* value and an odds ratio (OR) were defined. For variable correlation assessment, the parametric Pearson and non-parametric Spearman correlation tests were applied. Receiver operating characteristic (ROC) analyses were performed to assess the discriminatory power of hematological parameters for distinction between SARS-CoV-2 and influenza infections. Significance threshold for the results was defined as a *p* value below 0.05, corresponding to a 95% confidence interval (CI).

### 2.4. Ethics

The research respected the principles of the Declaration of Helsinki. Prior to hospitalization admittance, at least one legal guardian of the patients included in the study signed an informed consent form, which certified the approval of medical data use for scientific purposes, upon preservation of anonymity. Moreover, the research protocol was approved by the Ethics Committee of the County Emergency Clinical Hospital of Târgu Mureș County (approval no. 33386/15 January 2025).

## 3. Results

The two groups were similar in terms of mean age (2.61 ± 2.92 versus 2.54 ± 3.21, *p* = 0.23) and gender distribution (*p* = 0.93). Mean hospitalization duration was significantly longer in influenza infections (5.39 ± 5.28 versus 2.91 ± 2.27, *p* < 0.01) and PICU admission rates were superior within the influenza group (*p* < 0.01). Symptom comparison showed a significantly higher prevalence of cough (*p* < 0.01) and dysphonia (*p* = 0.03) in the influenza group (Table 1). On the other hand, cutaneous manifestations were more commonly encountered among children infected with SARS-CoV-2 (*p* = 0.03). In terms of digestive symptoms, a similar prevalence of abdominal pain and vomiting was identified between the two groups (*p* = 0.95 and *p* = 0.66). Despite this, children with SARS-CoV-2 infections presented a more than 4-fold higher risk of developing diarrhea (*p* < 0.01), but vomiting incidence was similar between the two study groups (*p* = 0.66, Table 1). Nevertheless, maximal recorded fever during admission was similar between the two groups (*p* = 0.43). Frequency of complications, such as respiratory distress and dehydration, was not significantly different between children with SARS-CoV-2 and those with influenza (*p* = 0.41 and *p* = 0.87, respectively). Febrile seizures were more commonly associated with influenza infections (*p* < 0.01), as well as modifications of chest X-ray suggestive of pneumonia diagnosis (*p* < 0.01, Table 1). These data have been provided through Table 1.

**Figure 1:**
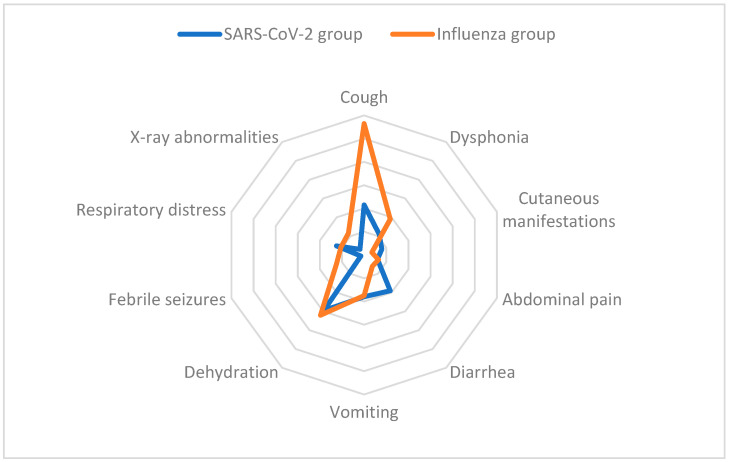
Spider graph for symptom distribution pattern between the two groups.

For a better representation of symptom distribution pattern between the two groups, a spider graph has also been provided (Figure 1). A clear predominance of cough is visible among the influenza group.

Comparative assessment of CBC, inflammatory and biochemistry parameters was conducted between the two study groups (Table 2). The Mann–Whitney test was applied for most of the comparisons conducted, with one isolated exception. No significant mean differences were found in leukocyte, lymphocyte, eosinophil, basophil, nor platelet counts (*p* = 0.56, *p* = 0.62, *p* = 0.15, *p* = 0.69 and *p* = 0.05, respectively, Table 2). Significantly higher neutrophil levels were found in children infected with influenza (5043 ± 3843/µL versus 4338 ± 4088, *p* = 0.02), whereas monocyte counts were more elevated in the SARS-CoV-2 group (1494 ± 1022 versus 1196 ± 806.7, *p* < 0.01). Mean MPV values were also higher in the same group with SARS-CoV-2 infection (9.83 ± 1.04 versus 9.50 ± 1.06, *p* = 0.01, Table 2). None of the red blood cell parameters presented significant variations between the two groups. Comparison of hematological derived parameters of inflammation, such as NLR and PLR, yielded no significant discrepancies (*p* = 0.08 and *p* = 0.87). However, LMR presented significantly higher values in the Influenza group (4.63 ± 3.78 versus 3.56 ± 2.79, *p* = 0.02, Table 2). Other inflammatory and biochemistry parameters, such as CRP, LDH, urea and creatinine, presented similar mean values between the two groups (*p* = 0.37, *p* = 0.75, *p* = 0.94 and *p* = 0.42, respectively). In addition, significant discrepancies were found for parameters of liver function, as higher mean liver enzyme levels were found in the SARS-CoV-2 group (*p* < 0.01 for both AST and ALT, Table 2).

A comparative evaluation of the same parameters was afterwards performed, after dividing the influenza group based on the type (A or B) of the infection. These results are presented in Table 3 and aid in a better understanding of the origin of mean difference between the three groups. For example, the significant difference in mean neutrophil levels is mainly based on the significantly higher values in the influenza B group (*p* = 0.03), which are also significantly higher than in the case of SARS-CoV-2 infections. The monocyte variation again yields significant results, with the highest values encountered in the SARS-CoV-2 group (*p* = 0.03). The highest Hgb and Htc levels were found in the influenza B group (*p* = 0.02), whereas LMR increase again constituted a marker of influenza infections, due to significant differences between the influenza B and SARS-CoV-2 groups (*p* = 0.04). The highest LDH levels were found in influenza b infections, whereas the most significant liver enzyme alterations where related to SARS-CoV-2 infections (Table 3).

ROC analysis of the inflammatory parameters and hematological parameters, which are shown before, to differ significantly between the two groups, is demonstrated in Table 4. As pictured in Table 4, cut-off values were obtained particularly for neutrophils, monocytes, MPV and LMR parameters, with moderate sensitivity and specificity levels, not exceeding 65.19% (*p* < 0.05). For the other parameters, no significant *p* values were obtained for the analyzed parameters (*p* > 0.05).

**Figure 2:**
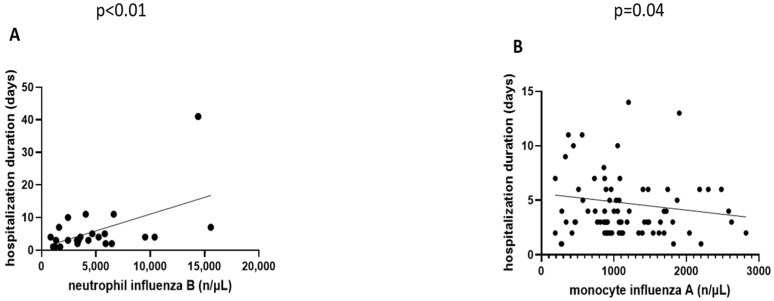
(**A**) Linear, ascending trend picturing a non-parametric Spearman correlation for the association between neutrophil levels and hospitalization duration in the influenza B study group. (**B**) Non-parametric Spearman correlation showcasing a linear, descending trend of monocyte levels in relation to hospitalization duration in the influenza A study group.

Linear correlation between parameters of inflammation, their hematological individual components, and hospitalization duration was further sought. Neutrophil levels positively correlated with hospitalization duration (*p* < 0.01, r = 0.46, 95% CI: 0.15–0.69) in the influenza B group, but did not present any association with the same parameter within the other two study groups. Monocyte values were inversely related to duration of hospital stay in the influenza A group (*p* = 0.04, r = −0.19, 95% CI: −0.38–−0.003). These results have been visually represented in Figure 2.

**Figure 3:**
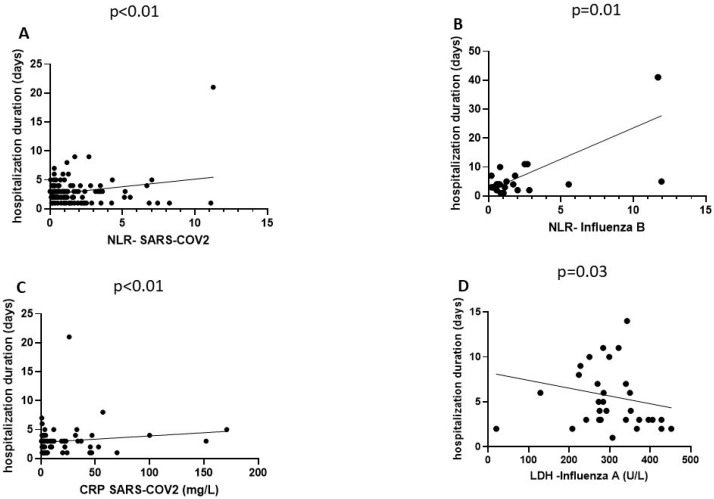
(**A**) Non-parametric Spearman correlation exhibiting a linear, ascending relationship between NLR and hospitalization duration in the SARS-CoV-2 group. (**B**) Non-parametric Spearman correlation shows a linear, descending trend between NLR and hospitalization duration in the influenza B group. (**C**) Non-parametric Spearman correlation for the linear, ascending association between CRP and hospitalization duration in the SARS-CoV-2 group. (**D**) Non-parametric Spearman correlation for the inverse, linear association between LDH and hospitalization duration in the influenza A group.

No significant correlations were found for hospitalization duration for either of the groups in regard to lymphocyte or platelet counts, PLR, or LMR. Significant linear association was found between NLR values and duration of hospital stay for both SARS-CoV-2 (*p* < 0.01, r = 0.23, 95% CI: 0.06–0.39) and influenza type B infections (*p* = 0.01, r = 0.41, 95% CI: 0.09–0.66). CRP values also presented an ascending trend in association with prolonged hospitalization duration for children infected with influenza A (*p* < 0.01, r = 0.37, 95% CI: 0.16–0.55). Nevertheless, an inverse correlation was found between LDH levels and hospitalization duration (*p* = 0.03, r = −0.33, 95% CI: −0.597–−0.007). The significant associations are pictured in Figure 3.

In addition, correlations between parameters of inflammation and the presence of respiratory distress were assessed. The significant results have been represented in Table 5. Leukocyte counts, LMR and CRP were predictive of the development of respiratory distress in children infected with influenza B. Lymphocyte counts and LMR were positively associated with the presence of respiratory distress in SARS-CoV-2 patients, whilst PLR was inversely associated with respiratory distress.

## 4. Discussion

SARS-CoV-2, influenza and respiratory syncytial virus (RSV) infections are responsible for potentially severe, complicated respiratory tract infections, which require hospitalizations and even ventilatory support. A nationwide study performed on a Singaporean adult population concluded that RSV determines the most severe forms of respiratory tract infections, requiring prolonged hospitalizations, but comparable to disease courses found in patients who have not received booster vaccination against SARS-CoV-2 [15]. As a matter of fact, vaccination against SARS-CoV-2 yielded lower likelihood of hospital and ICU admission rates and was positively associated with immunization against influenza in the same individual [16]. Age-dependent comparisons led to different results, as some studies have shown, for example, that the Omicron-predominant wave of SARS-CoV-2 infections yielded higher mortality rates than influenza in the elderly, but not at young ages [17]. One comparative study conducted in Saudi Arabia, analyzing the behavior of pediatric SARS-CoV-2, influenza and RSV infections, found the longest ICU hospitalization duration for patients infected with influenza type B. These results were traced back to the low number of influenza B cases and the lack of mandatory vaccination policy for influenza. Significantly higher hospitalization durations were also found for RSV, a viral infection, which also required overall a longer hospital stay [18]. In contrast, one study enrolling both children and adults, conducted in the United States of America during the 2022–2023 autumn-winter season, revealed compellingly higher hospitalization rates for SARS-CoV-2 infections than for influenza. Within the study, regardless of age group, number of hospitalizations for SARS-CoV-2 was significantly higher than the number of influenza-related admissions. Whilst most of the SARS-CoV-2 related hospitalizations were composed of patients with underlying disorders, an important percentage of children aged 0–5 years (more than 35%) and aged 6–17 years (more than 25%) had no chronic medical conditions [19]. In our study, we found a significantly higher hospitalization duration for influenza infections, as well as higher PICU admission rates in the same category. These results might have also been influenced by the exclusion of SARS-CoV-2 infections complicated by multisystem inflammatory syndrome.

After ending the zero COVID policy in countries like China, a resurgence of respiratory tract infections caused by miscellaneous viral agents was reported. These encompassed high positivity rates for RSV, human rhinovirus (HRV) and human parainfluenza virus (HPIV) [20]. Literature data investigating the clinical manifestations and paraclinical data particularities of viral respiratory tract infections before and after the pandemic have surfaced recently. For example, influenza A-infected children presented more frequent respiratory symptoms and complications such as myositis after the pandemic as opposed to the timeframe before 2020. Furthermore, a trend towards WBC decrease and liver enzyme increase was seen more frequently in the same category of patients after the pandemic [21]. In our study, we identified higher liver enzyme values in SARS-CoV-2 patients than in the two influenza categories. In other studies, abnormal liver enzymes were commonly found in both types of infections during hospitalization and were regarded as markers of poor outcome, reflecting disease severity [22,23]. In a study conducted during a pandemic outburst of influenza A/H1N1 infections, liver enzyme elevation was associated with hypoxia and was more pronounced in patients infected with the aforementioned viral strain as opposed to seasonal influenza infections [24]. Nevertheless, in pediatric acute respiratory tract infections, liver inflammation and subsequent abnormal liver tests seem to affect around 10% of children and adolescents, without relation to the medication used [25].

The clinical picture is miscellaneous in SARS-CoV-2 infections, including a wide category of symptoms even in children. Fever and cough are usually found in influenza, and are also symptoms of SARS-CoV-2, whereas gastrointestinal symptoms rarely manifest in pediatric influenza infections, but are more prevalent with SARS-CoV-2 [4]. In particular, the Omicron variant has led to more frequent digestive symptoms in infants in Romania, as highlighted by one pediatric study [26]. In similar fashion, in our study we found a significantly higher prevalence of diarrhea in the SARS-CoV-2 group. Nevertheless, we also identified a significantly higher prevalence of febrile seizures in association with influenza infections, which is in line with previous studies which categorized severe influenza infections, especially influenza type A, as risk factors of febrile seizures [27].

A review investigating the particular evolution of hematological parameters in pediatric cases of SARS-CoV-2 infection showed that normal leukocyte levels are commonly encountered, with leukopenia representing the most frequent abnormality of white blood cell levels. Lymphopenia seems to be less usual in adults, probably due to the less severe forms of the disease in this particular age group. Furthermore, anemia and hypercoagulability can be suggestive of a multisystemic inflammatory syndrome [28]. Still, some studies also show that hematological parameter variations in pediatric SARS-CoV-2 infections vary in relation to age group, with miscellaneous discrepancies from healthy subjects at neonatal, infant, childhood and adolescence stages [29]. One pediatric study showed that complete blood count changes, such as for leukopenia, lymphopenia and thrombocytopenia, were more particular of influenza infections. On the other hand, significant monocytosis seems to be common to both viral infections [1]. Furthermore, significantly higher leukocyte, neutrophil and platelet numbers, as well as important elevations in NLR values, were seen in children with SARS-CoV-2, when compared to those with influenza [30]. A comparative pediatric study enrolling children with influenza, a group with other viral respiratory tract infections and a control group showed that, particularly at very young ages, NLR, LMR and lymphocyte levels can help in the distinction of influenza from other viral infections [31]. In our study, we identified neutrophil and monocyte levels as discriminators between SARS-CoV-2 and influenza. These findings confirm the monocyte activation previously described in SARS-CoV-2 infections, which is similar to that found in other infectious processes. Hematologically derived ratios also seem to play an important role in differential diagnosis and outcome prediction, particularly for bacteremia and influenza infections. In children, NLR seems to aid in the distinction between *Mycoplasma pneumoniae* and influenza A [32]. Moreover, in pediatric patients diagnosed with croup and SARS-CoV-2 infections, NLR correlated with the Westley croup score and was predictive of ICU admission [33]. Although NLR did not differ significantly between our study groups, our results also show the importance of NLR for adverse outcome prediction, through its positive correlation with hospitalization duration in both SARS-CoV-2 and influenza B groups.

Within our study, we found no significant differences between the study groups in platelet counts, nor in PLR values. Previous studies conducted on pediatric populations have also failed to identify any significant differences in platelet and PLR levels between SARS-CoV-2 and influenza infections, although both parameters seem to present significant alterations from healthy controls in both infections [31,34,35,36]. Still, we found a significant correlation between PLR and the presence of respiratory distress in the SARS-CoV-2 cohort. A particular interesting finding of the current research was the importance of MPV in the distinction of SARS-CoV-2 from influenza infections, as compellingly higher values were found in children infected with SARS-CoV-2 when comparing mean differences. Some previous studies reported no important variations in MPV levels between SARS-CoV-2 and influenza infections, whereas others have obtained similar results to ours [11,35,37]. Nevertheless, MPV has been regarded as a prognostic marker of diseases with systemic inflammation such as SARS-CoV-2, which can be accompanied by thrombotic processes [38]. As MPV increase seems to be more specific to SARS-CoV-2 infections than other viral respiratory infections in children, it has been proposed as part of a conjoint diagnostic algorithm, together with other platelet indices, for the early triage of SARS-CoV-2 infections [39].

In our study, we have particularly chosen to investigate how LMR performs in distinguishing the two viral infections due to previous evidence, which has shown that this parameter poses a high sensitivity and specificity for the correct differentiation of influenza viruses form other viral infections exhibiting similar symptoms, when applied to a cohort of children with similar symptoms [40]. Previous data asserted that LMR presents a greater increase in relation to influenza infections in children under 6 years of age, as opposed to healthy subjects and those with other viral respiratory tract infections [31]. In similar fashion, within our study, LMR was significantly higher in both influenza groups in comparison to the SARS-CoV-2 group, without obvious differences between influenza A and influenza B infections. The same parameter particularly correlated with the presence of respiratory distress for both SARS-CoV-2 and influenza B cohorts. This finding is in line with previous suppositions of increase in LMR values as predictors of a more adverse outcome, as one study has showed that a cut-off level of 2 for LMR could be an indicator of hospitalization requirement in influenza children [41]. Therefore, LMR should be incorporated as an accompanying parameter of routine laboratory testing in children with SARS-CoV-2 and influenza infections with complicated courses.

In the adult population, stronger evidence emerged upon the application of hematological parameters, as result of the higher number of studies available in the literature. For example, one study concluded that NLR and PLR can distinguish healthy subjects from adults infected with influenza B, whereas NLR, PLR, LMR, leukocyte count and CRP can be used for patient follow-up [42]. NLR was regarded as an early predictor of critical illness in patients aged over 50 years infected with SARS-CoV-2, but not with influenza or respiratory syncytial virus [43,44]. Although several studies have demonstrated that PLR and LMR are also predictors of severe outcome, being able to predict ICU admission requirement and progression to acute respiratory distress syndrome (ARDS), one meta-analysis showed that NLR still had the highest predictive value for severe SARS-CoV-2 infections of all hematological ratios [45]. Moreover, high neutrophil monocyte ratios have also been associated with severe disease course in SARS-CoV-2 adult infections [46]. Through our study, we have also tried to identify how the hematological parameters perform in predicting hospital stay and the development of respiratory distress, as the importance of CBC parameters in predicting complications had not been investigated in depth in pediatric populations. Amongst others, we have found significant correlations between NLR, LMR and both hospitalization duration and respiratory distress for both SARS-CoV-2 and influenza B infections.

In terms of inflammatory markers, we found no significant disparities between the two groups for CRP nor LDH. A systematic review of the literature concluded that SARS-CoV-2 infections cause less increase in CRP and procalcitonin values when compared with influenza [47]. Another systematic review concluded that the two infections do not cause significantly different variations in CRP and procalcitonin values, but found a contrasting increase in LDH values for influenza infections in children [48].

The current study has brought into light clinical differences and paraclinical particularities of two commonly encountered respiratory tract infections and included both a pandemic and a post-pandemic timespan. Still, several limitations can impact the results obtained within this study. First of all, the retrospective character of the study, the limited number of subjects included and, in particular, the small number of influenza B infections encountered could have influenced the reported outcomes. Secondly, the decision to exclude SARS-CoV-2 infections complicated by multisystemic inflammatory syndrome could have led to an underestimation of SARS-CoV-2-related severity and complications. Nevertheless, multisystemic inflammatory syndrome inclusion would have led to significant changes in hematological parameters and unrealistic disparities in comparison with influenza infections. A review focusing on pediatric studies has shown that certain hematological abnormalities are more frequently encountered in SARS-CoV-2-associated multisystem inflammatory syndrome as opposed to the classical clinical courses of SARS-CoV-2 infections, such as anemia, thrombocytopenia or neutrophilia [28]. Hence, multisystemic inflammatory syndrome represents a complication of SARS-CoV-2 with particular hematological changes, which cannot be compared to those triggered by other viral infections with respiratory tropism.

The results obtained emphasize how certain parameters of the CBC along with particular derived ratios might aid in the differentiation of SARS-CoV-2 from influenza infections in children, and in possible outcome prediction. Hence, in children presenting to a hospital facility, a CBC could represent a screening method for assessing hospitalization duration and the development of respiratory distress, particularly through determination of parameters such as MPV, LMR, NLR or total leukocyte and lymphocyte counts. Nevertheless, neutrophils, monocytes, as well as MPV and LMR, two parameters not commonly considered when analyzing a CBC, seem to be able to discriminate between SARS-CoV-2 and influenza in children and should constitute one of the primary focuses in clinical practice. However, the findings obtained in our study require further validation in larger-scaled, prospective, multicentric pediatric studies.

## 5. Conclusions

SARS-CoV-2 and influenza infections produce similar clinical pictures in children, but with a particular higher incidence of digestive symptoms in the case of SARS-CoV-2 and a more frequent occurrence of febrile seizures for influenza infections. In our study, we found a significantly higher duration of hospital stay and a higher PICU admission rate for pediatric influenza infections. In terms of CBC parameters, monocyte levels, together with LMR, might particularly aid in the differentiation of the two infections. Several parameters of the CBC corelated with hospital length of stay and with respiratory distress. A peculiar association between NLR and hospitalization duration as well as between LMR and the presence of respiratory distress was found for both SARS-CoV-2 and influenza B infections. For influenza B infections, CRP and leukocyte levels also correlated with respiratory distress, but, interestingly, for influenza A only one significant association was found, between LDH and hospitalization duration. As the current study included a relatively limited population sample, further studies, enrolling larger pediatric study groups, could confirm/refute our findings and facilitate a better understanding of the behavior and particular characteristics of influenza and SARS-CoV-2 infections in children.

## Figures and Tables

**Table 1:** Comparison of symptom and complication frequency between the two study groups.

Parameter		SARS-CoV-2 Group (n = 129)	Influenza Group (n = 137)	*p* Value
Gender (n)	female	53	57	*p* = 0.93, (95% CI: 0.59–1.60)
	male	76	80	
Age (years)		2.61 ± 2.92	2.54 ± 3.21	*p* = 0.23
Hospitalization duration (days)		2.91 ± 2.27	5.39 ± 5.28	*p* < 0.01 *
PICU admission	Yes	0	7	*p* < 0.01 *, (95% CI: 0.00–0.56)
	No	129	130	
Cough (n)	Yes	43	113	*p* < 0.01 *, (95% CI: 0.06–0.18)
	No	86	24	
Dysphonia (n)	Yes	22	38	*p* = 0.03 *, (95% CI: 0.29–0.94)
	No	107	99	
Cutaneous manifestations (n)	Yes	16	7	*p* = 0.03 *, (95% CI: 1.06–6.77)
	No	113	130	
Abdominal pain (n)	Yes	12	13	*p* = 0.95, (95% CI: 0.41–2.22)
	No	117	124	
Diarrhea (n)	Yes	38	12	*p* < 0.01 *, (95% CI: 2.18–8.49)
	No	91	125	
Vomiting (n)	Yes	36	35	*p* = 0.66, (95% CI: 0.65–1.94)
	No	93	102	
Dehydration (n)	Yes	59	64	*p* = 0.87, (95% CI: 0.59–1.56)
	No	70	73	
Febrile seizures (n)	Yes	3	25	*p* < 0.01 *, (95% CI: 0.03–0.34)
	No	126	112	
Respiratory distress (n)	Yes	25	21	*p* = 0.41, (95% CI: 0.70–2.50)
	No	104	116	
X-ray abnormalities (n)	Yes	6	23	*p* < 0.01 *, (95% CI: 0.09–0.61)
	No	123	114	

Legend: *—*p* < 0.05; CI—confidence interval; PICU—pediatric intensive care unit.

**Table 2:** Comparison of hematological, inflammatory and biochemistry parameters between the two study groups.

Parameter	SARS-CoV-2 Group (n = 129)	Influenza Group (n = 137)	*p* Value
Leukocytes (n/µL)	10,504 ± 5940	10,879 ± 5807	0.56
Neutrophils (n/µL)	4338 ± 4088	5043 ± 3843	0.02 **
Lymphocytes (n/µL)	4475 ± 3204	4518 ± 3610	0.62
Monocytes (n/µL)	1494 ± 1022	1196 ± 806.7	<0.01 **
Eosinophils (n/µL)	121.1 ± 169.2	115.5 ± 189.2	0.15
Basophils (n/µL)	37.29 ± 37.76	36.57 ± 29.12	0.69
Platelets (n/µL)	319,609 ± 134,608	305,570 ± 144,679	0.05
MPV (fL)	9.83 ± 1.04	9.50 ± 1.06	0.01 *^,^ **
Erythrocytes (n × 10^6^/µL)	4.14 ± 0.72	4.29 ± 0.60	0.16
Hgb (g/dL)	12.09 ± 8.14	11.58 ± 1.76	0.13
Htc (%)	32.66 ± 5.79	33.24 ± 5.71	0.14
MCH (pg)	27.95 ± 5.63	28.35 ± 3.97	0.15
MCV (fL)	80.18 ± 8.91	78.54 ± 7.68	0.27
NLR	1.59 ± 2.04	2.24 ± 2.85	0.08
PLR	113.5 ± 96.05	121.9 ± 120.1	0.87
LMR	3.56 ± 2.79	4.63 ± 3.78	0.02 **
CRP (mg/L)	18.21 ± 34.79	18.37 ± 28.82	0.37
LDH (U/L)	349.3 ± 132.3	329.1 ± 155.4	0.75
Creatinine (mg/dL)	0.32 ± 0.12	0.33 ± 0.19	0.94
Urea (mg/dL)	18.90 ± 9.88	19.31 ± 9.14	0.41
AST (U/L)	47.22 ± 17.2	46.03 ± 34.20	<0.01 **
ALT (U/L)	26.73 ± 22.46	20.61 ± 13.99	<0.01 **

Legend: *—unpaired t test with Welch’s correction was applied; **—*p* < 0.05; ALT—alanine aminotransferase; AST—aspartate aminotransferase; CRP—C reactive protein; Hgb-Hemoglobin; Htc—Hematocrit; LDH—lactate dehydrogenase; LMR—lymphocyte to monocyte ratio; MCH—mean corpuscular hemoglobin; MCV—mean corpuscular volume; MPV—mean platelet volume; n—number; NLR—neutrophil to lymphocyte ratio; PLR—platelet to lymphocyte ratio.

**Table 3:** Comparative analysis of hematological, inflammatory and biochemistry parameters among SARS-CoV-2 group and the two influenza infection groups.

Parameter	SARS-CoV-2 Group (n = 129)	Influenza A Group (n = 101)	Influenza B Group (n = 36)	*p* Value
Leukocytes (n/µL)	10,504 ± 5490	10,381 ± 4980	12,247 ± 7552	0.57
Neutrophils (n/µL)	4338 ± 4088	4717 ± 3545	5941 ± 4498 ^a^	0.03 *
Lymphocytes (n/µL)	4475 ± 3204	4447 ± 3587	4715 ± 3715	0.85
Monocytes (n/µL)	1494 ± 1022	1138 ± 621 ^b^	1355 ± 1173	0.03 *
Eosinophils (n/µL)	121.1 ± 169.2	98.99 ± 137.6	160.8 ± 284.9	0.33
Basophils (n/µL)	37.29 ± 37.76	37.07 ± 26.31	35.19 ± 36.12	0.28
Platelets (n/µL)	319,609 ± 134,608	288,960 ± 116,498 ^b^	351,250 ± 198,020	0.10
MPV (fL)	9.83 ± 1.04	9.49 ± 1.11 ^b^	9.53 ± 0.92	0.03 *
Erythrocytes (n × 10^6^/µL)	4.14 ± 0.72	4.23 ± 0.57	4.45 ± 0.65 ^a^	0.06
Hgb (g/dL)	12.09 ± 8.14	11.37 ± 1.86	12.14 ± 1.30 ^a,c^	0.02 *
Htc (%)	32.66 ± 5.79	32.77 ± 5.60	34.52 ± 5.88 ^a,c^	0.02 *
MCH (pg)	27.95 ± 5.63	28.13 ± 3.94	28.94 ± 4.04	0.30
MCV (fL)	80.18 ± 8.91	78.11 ± 7.65	79.72 ± 7.74	0.47
NLR	1.59 ± 2.04	2.22 ± 2.77	2.29 ± 3.09	0.15
PLR	113.5 ± 96.05	119.8 ± 119	127.5 ± 124.8	0.75
LMR	3.56 ± 2.79	4.63 ± 3.98	4.63 ± 3.24 ^a^	0.04 *
CRP (mg/L)	18.21 ± 34.79	20.12 ± 31.37	17.60 ± 23.34	0.95
LDH (U/L)	349.3 ± 132.3	291.5 ± 89.36	404.3 ± 223 ^a,c^	0.01 *
Creatinine (mg/dL)	0.32 ± 0.12	0.35 ± 0.20	0.29 ± 0.14	0.15
Urea (mg/dL)	18.90 ± 9.88	19.48 ± 10.07	18.93 ± 6.84	0.71
AST (U/L)	47.22 ± 17.2	43.36 ± 25.49 ^b^	40.31 ± 14.93 ^a^	<0.01 *
ALT (U/L)	26.73 ± 22.46	19.51 ± 9.91 ^b^	23.38 ± 20.94 ^a^	<0.01 *

Legend: ^a^—*p* < 0.05 for SARS-CoV-2 versus Influenza B group; ^b^—*p* < 0.05 for SARS-CoV-2 versus Influenza A group; ^c^—*p* < 0.05 for Influenza A versus Influenza B group; *—*p* < 0.05; ALT—alanine aminotransferase; AST—aspartate aminotransferase; CRP—C reactive protein; Hgb—Hemoglobin; Htc—Hematocrit; LDH—lactate dehydrogenase; LMR—lymphocyte to monocyte ratio; MCH—mean corpuscular hemoglobin; MCV—mean corpuscular volume; MPV—mean platelet volume; n—number; NLR—neutrophil to lymphocyte ratio; PLR—platelet to lymphocyte ratio.

**Table 4:** ROC analysis of hematological and inflammatory parameters for the differentiation between SARS-CoV-2 and influenza infections.

Parameter	AUC (95% CI)	Cut-Off Value	Sensitivity (95% CI)	Specificity (95% CI)	*p* Value
Neutrophil (n/µL)	0.58 (0.51–0.65)	>3325	58.52% (50.08–66.48%)	57.36% (48.74–65.56%)	0.02 *
Monocyte (n/µL)	0.59 (0.52–0.66)	<1105	60.74% (52.32–68.57%)	58.14% (49.51–66.30%)	<0.01 *
MPV (fL)	0.59 (0.52–0.66)	<9.75	61.36% (52.85–69.24%)	56.91% (48.08–65.32%)	<0.01 *
NLR	0.56 (0.49–0.63)	>0.74	65.19% (56.63–72.70%)	43.31% (35.01–52.00%)	0.08
PLR	0.50 (0.43–0.57)	>89.02	51.11% (42.77–59.40%)	57.48% (48.79–65.73%)	0.87
LMR	0.58 (0.51–0.65)	>3.41	51.11% (42.77–59.40%)	66.14% (57.55–73.79%)	0.02 *
CRP (mg/L)	0.51 (0.42–0.59)	>5.50	52.38% (42.91–61.68%)	53.62% (41.98–64.89%)	0.81
LDH (U/L)	0.51 (0.40–0.61)	<299.5	45.61% (33.37–58.41%)	50.88% (38.26–63.38%)	0.82

Legend: *—*p* < 0.05; ALT- alanine aminotransferase; AUC—area under the curve; CRP—C reactive protein; LDH—lactate dehydrogenase; LMR—lymphocyte to monocyte ratio; MPV—mean platelet volume; NLR—neutrophil to lymphocyte ratio; PLR—platelet to lymphocyte ratio.

**Table 5:** Significant correlations between parameters of inflammation and the presence of respiratory distress.

Parameter	Study Group	Non-Parametric Spearman Correlation (r, 95% CI)	*p* Value
Leukocyte count (n/µL)	Influenza B	r = 0.33 (95% CI: −0.003–0.603)	0.04
Lymphocyte count (n/µL)	SARS-CoV-2	r = 0.18 (95% CI: 0.01–0.35)	0.03
PLR	SARS-CoV-2	r = −0.20 (95% CI: −0.37–−0.02	0.01
LMR	SARS-CoV-2	r = 0.20 (95% CI:0.02–0.37)	0.02
LMR	Influenza B	r = 0.37 (95% CI: 0.04–0.63)	0.02
CRP	Influenza B	r = 0.40 (95% CI:0.009–0.691)	0.03

## Data Availability

The data presented in this study are available on request from the corresponding author.
